# 

**Published:** 1893-01-14

**Authors:** 


					u. 1893. THE HOSPITAL,
Suiwu- - CONTENTS.
of Girls at Schools
213
toecUca? ^?Pi?s ?
-Taken on Trust
244
Taken on Trnst -
WIIR "1*tory from the Earliest Times.
By E. T.
^iTHiwrf? ory from the Earl
Boh&??. . M;A., M.B. Oxon.
XXXVIII. ?The Arbo-
*r SehoWinlT M-B- Ox<
5?* Boovl ? M Mertioine
245
Book??EeJi?1- (1) Me<iioine
-"Keep your Month Shut " -
246
?3WtKlST ^ons*?" Keep your Mouth Shut "
246
St Ti u"nio-
iw?*H*L?MF;w's Hospipai.
?The Treatment of Strumous
DiW U1A,MKW S HOSP
. 251
Huu Botit T Knee-j^"
RoUthJ iNFIRMABT.
?The Treatment of Tonsillitis
253
Hotal anfirmaet.?'The Treatmen
Ctrio niHEEN Hospital, Liverpool.
-The Treatment of
n T ^vuamiisb
jOMtao Ulcer
253
Tai i> Ulcer ?
rPhoJfIlACTITI0*?ns'
-"The Chemistry and
Thfiro 5ITI0KBns'
neraPeutics ol Gout'
254
Water Supply and Waste
247
Everybody's Page
248
Annotations.-
-The DrnnVard's Deadlock?What shall we do with
crar Dea 1 ??The Indispansable " Spado! "
249
Notes and News ?
250
The Institutional Workshop ?
Cocking by Gas. II.
255
?The Wametord Hospital,Leamington
256
Construction Notes
257
Literary Iktblliqence
257
New hospitals.-
-Opening ef the Royal Bye Ho3pitaI,
Southwark
258
Scraps and Gleanings  . 258
The Student of Medicine -Appointments?Vacancies.
If EXTRA SUPPLEMENT.?THE NUBSINQ MIRROR.
Hew JJi ^.T ?C A BUi'I'IiJSJa.JaJMJi
dayglo??-*?16 Wursingf World.?Nurses' Christmas Holi-
?^ovelinfio?^ o temE?OTitio?Our Endowed Bed?Loaned
Hand-1 tr "jocessful Sale at Shoebnryness?With Empt r
" Outbb " Xf"<. Benevolenoe ? A Welsh Minister's
?i?v Australian wa r Miaericordite, Dublin?Pleased Patients?An
"he Kuraes' Home
. Indian Oi?oflent of Children by Gymnastics. IV.?
^Polataienta
cxvii
cxviii
Minor Appointments - cxvii?
Wants and Workers cxTiif
Christmas Festivities oxvii1
The International Nursing1 Congress, Chicago - - oxix
Everybody's Opinion.?"Nurses and their Fees '?Oar En-
dowed Bod?Not a Policy Holder - cxix
Presentations oxx
Where to Go oxx
Notes and Queries oxx

				

